# Medicine

**Published:** 1920-03

**Authors:** J. A. Nixon


### Progress of the Medical Sciences.

### MEDICINE.

**Cerebro-Spinal Meningitis.**—A review of this disease in the light of recent additions to our knowledge.

*Epidemiology.*—The season of maximum prevalence is February–March, that of minimum prevalence August–September. The disease is not spread as a meningitis, what is spread is the meningococcus in the secretions of the nose and throat. The proportion of individuals so little resistant to the meningococcus as to develop meningitis is very low as compared with those who remain unaffected or develop only a minor illness (Parkes and Kenwood [1]). The prevalence of " colds " in the population appears to favour the outbreak of cerebro-spinal meningitis, but the " cases " need not have had colds or naso-pharyngitis (Rolleston [2]). " Carriers " do not often develop the disease, but they usually show the same type of meningococcus as the cases prevalent. A recrudescence of the disease shows great increase in the number of carriers, but non-contacts often show a higher carrier rate than those supposed to be contacts (Flack and Glover [3]). There are three possibilities to account for an individual case—

1. A " positive," *i.e.* a carrier, suddenly loses his resistance and develops infection of the cerebro-spinal system.

2. A " negative " becomes infected from an " exalted " strain derived directly from a case.

3. A " negative " becomes infected from a " positive," and instead of " carrying " the meningococcus he succumbs to cerebro-spinal meningitis.

As a practical point, it is clear that out of a large number of individuals isolated as carriers none will develop cerebro-spinal meningitis except perhaps as a rare exception (Fildes and Baker [4]). In an epidemic in Schwerin (1915–16) 10,000 examinations were made, and the " carriers " isolated. No carrier contracted cerebro-spinal meningitis. Soldiers from the front became carriers in a few days, but while the number of " carriers " increased the number of cases of meningitis diminished. The publication of this report was prohibited as contrary to accepted teaching and common knowledge (Much [5]).

Overcrowding is probably important, and Glover found a high carrier rate associated with overcrowding in sleeping quarters. Where the distance between the beds was increased the carrier rate fell in a few weeks from over 20 per cent. to the normal 5 per cent. (Gordon [6]). Yet Leishman [7] found that it was difficult to trace individual cases occurring in our armies in France to overcrowding, or to trace any connection between cases which were mostly sporadic. Cases of cerebro-spinal meningitis are not carriers *before* the onset, therefore they cannot have infected the contacts who are found positive *at* the onset. The "case" is as often as not free from cocci in the throat a short time after the onset, and, further, is seldom at any time so heavily infected or so persistent as an ordinary carrier. The declared "case" has very little to do with the propagation of cerebro-spinal meningitis, and is less likely to spread infection than a "carrier." The disease, then, is more or less an accidental by-product of a throat epidemic, during the course of which an occasional individual is found who succumbs to cerebro-spinal meningitis. A "case" occurs because in the vicinity of a susceptible individual there is a fairly high rate of positive carriers (Fildes and Baker [4]).

*Bacteriology.*—Gordon [6] has described four types which can be differentiated by agglutination and absorption tests. There is evidence of close relationship between Types I. and III. (Group A), and between II. and IV. (Group B). I. and IV. are the more highly specialised (in their agglutination reaction) of each pair. Group A corresponds with *meningococcus*, Group B with *para-meningococcus*. II. and IV. are commonest in the throat. I. and III. [2] which are less common, are more pathogenic, they only appear in epidemic numbers in the winter, fading out in the spring. It has been observed that Group A (I. and III.) favour the meninges; Group B (II. and IV.) cause septicæmia and extra-meningeal metastases. But Rolleston has seen Type II. give rise to severe meningitis. Before the war 96 per cent. of cases were Group A; during the war Group B became equally if not more common.

*Treatment.*—The following rules should be adhered to in regard to serotherapy:—

1. The diagnosis is made by lumbar puncture. If the fluid is turbid or under pressure give serum immediately of the prevailing type or polyvalent. The amount of serum should be equal to the amount of fluid withdrawn, not less than 20 c.c. The serum should be warmed to body temperature. After injection the foot of the bed should be raised.

2. The type of meningococcus should be determined and the appropriate serum given. Type II. serum contains less anti-endotoxin than Type I., and should therefore be given twice a day.

3. Serum should be given at least daily till the danger is past. It should be repeated daily for four days whether improvement follows or not. It should be discontinued only when the temperature remains normal, and should be repeated on any recrudescence of temperature. The prospect of serum proving successful is materially diminished if its administration is not begun before the sixth day of illness.

*Treatment of Carriers.*—Gordon[6] advises the use of a steam atomiser (Levick's or Hine's pattern) with a solution of 1 per cent. zinc sulphate or 2 per cent. Chloramine. The sprayed air must be inhaled vigorously for five or ten minutes. Chronic carriers may be treated by applying Acriflavine (1 in 1,000) several times daily. Such cases should always be examined by a rhinologist for abnormalities of the nasal passages and accessory sinuses. It is impossible to estimate the real value of treatment in the cure of carriers, as it was found in the Navy that one-third of the carriers discovered amongst recruits became cured immediately upon entry, whatever the treatment applied to them.

*Clinical Characters.*—Rolleston[2] classifies the disease into two main groups: the systemic and the meningitic, with a further sub-division according to the clinical course. For comparison Rolleston's classification is shown below together with Osler's and Rose Bradford's.[8]

| *Rolleston.* | *Osler.*[10] | *Rose Bradford.*[8] |
|---|---|---|
| 1. Fulminating. | *a.* Malignant form (fulminating or apoplectic). | 1. Meningitic (cerebral or spinal). |
| 2. Ordinary acute. | *b.* Ordinary form. | 2. Typhoidal or pneumonic. |
| 3. Abortive. | *c.* Anomalous forms. | 3. Anomalous symptoms. |
| 4. Chronic. | 1. Abortive type. | 4. Resembling perfectly another disease, *e.g.* rubeola, rheumatism, myalgia, trench fever or nephritis. |
| 5. Septicæmic. | 2. Intermittent or pyæmic. | |
| 6. Encysted or loculated meningitis. | 3. Chronic. | |

The general meningitic features may be summarised thus: intense headache, pain and stiffness in muscles especially those of the neck, head-retraction, fever, vomiting, delirium, and later coma. Positive Kernig's sign, strabismus and photophobia. Various rashes are met with: rose spots, papules and petechiæ. Herpes labialis is common (25 per cent. of cases). Foster[9] mentions two points of special importance in early diagnosis: Kernig's sign and retention of urine, or difficulty in micturition.

REFERENCES.

[1] Parkes and Kenwood, *Hygiene and Public Health*, Sixth Edition, 1917 p. 510.
[2] Rolleston, *Brit. M. J.*, 1919, i. 640 *et seq.*
[3] Flack and Glover, quoted by Gordon, *vide infra.*
[4] Fildes and Baker, *Med. Research Comm. Spec. Report Series.* No. 17.
[5] Much, *München. med. Wchnschr*, 1919, lxvi. 52; quoted in *Med. Suppl.*, 1919, ii. 182.
[6] Gordon, *Med. Bull. Am. Red Cross Soc. in France*, 1918, i. 342.
[7] Leishman, *Ibid.*, p. 350.
[8] Rose Bradford, *Ibid.*, p. 352.
[9] Foster, *Ibid.*, p. 346.
[10] Osler, *Principles and Practice of Medicine*, Eighth Edition, 1912.

J. A. NIXON.

